# The Intramuscular Versus the Intrathecal Route in the Treatment of Tetanus by the Injection of Antitoxin

**Published:** 1918-01

**Authors:** 


***The Intramuscular versus the Intrathecal Route in the Treatment of Tetanus by the Injection of Antitoxin.*** By Sir David BRUCE, Surgeon-General, R. A. M. C. Abs. from the *Lancet,* May 5, 1917.

B., on behalf of the Tetanus Committee, challenges the statements made by Leishman and Smallman against the value of the intrathecal method of injection and in favor of the intramuscular route (see p. 189). B. supports the Committee's contention that the intrathecal route is the most reliable, by tabulating results from a series of experiments performed upon monkeys by Prof. C. S. Sherrington. There were 30 animals in the group. 12 were treated intramuscularly and all died. Of 18 treated intrathecally 13 recovered. To strengthen the conclusions thus indicated, B. notes that in several cases treated intrathecally a higher dose of toxin was administered than in any of those cases treated by the intramuscular route. In some of the intrathecal experiments, too, a smaller amount of antitoxin was injected than in any of the intramuscular ones.

